# MiR-21 and let-7 cooperation in the regulation of lung cancer

**DOI:** 10.3389/fonc.2022.950043

**Published:** 2022-09-29

**Authors:** Jinquan Bai, Zhenzhou Shi, Shuting Wang, Hong Pan, Tong Zhang

**Affiliations:** Department of Radiology, The Fourth Affiliated Hospital of Harbin Medical University, Harbin, China

**Keywords:** lung cancer, miR-21, let-7, K-ras, cooperative regulation

## Abstract

**Background:**

Lung cancer occurs and develops as a result of a complicated process involving numerous genes; therefore, single-gene regulation has a limited therapeutic effect. We discovered that miR-21 expression was high in lung cancer tissues and cells, whereas let-7 expression was low, and it is unclear whether their combined regulation would be superior to therapy involving single regulation. The goal of our research was to investigate this situation and the regulatory mechanism that exists between these genes.

**Methods:**

To regulate the levels of miR-21 and let-7 in these two types of lung cancer cells, we transfected miRNA mimics or inhibitors into A549 and H460 cells. Lung cancer cells were tested for proliferation, apoptosis, migration, and invasion. The results were verified using a Western blot and a qRT-PCR assay. Bioinformatics was used to investigate their potential regulatory pathways, and luciferase assays were used to confirm the binding sites.

**Results:**

The expression of miR-21 was increased and that of let-7 was decreased in lung cancer tissues and cells compared with paracancerous tissues and normal lung cells (*p* < 0.01). Tumor cells were inhibited by downregulation of miR-21 and upregulation of let-7, and cooperative regulation showed a better effect. Upregulation of miR-21 and downregulation of let-7 promoted tumor cells, and this tumor-promoting effect was amplified by cooperative regulation. MiR-21 regulated lung cancer cells directly *via* the Wnt/-catenin pathway, and let-7 exerted its effects *via* the PLAG1/GDH1 pathway. MiR-21 and let-7 cooperated to regulate lung cancer cells *via* the K-ras pathway.

**Conclusions:**

The effect of cooperative regulation of miR-21 and let-7 on lung cancer is greater than that of a single miRNA. MiR-21 and let-7 are important differentially expressed genes in lung cancer that are regulated by the K-ras pathway. As a result, for multigene lung cancer, the cooperative regulation of two miRNAs will provide a new target and direction for lung cancer treatment in the future.

## Introduction

Mutations in coding genes and noncoding gene disorders are the primary causes of lung cancer occurrence and progression (1). The targeted drug therapy strategy for lung cancer is primarily aimed at the type of gene mutation (2), but drug resistance caused by frequent gene mutation has always been a difficult problem in lung cancer treatment (3, 4). As a result, the regulation of noncoding genes involved in lung cancer treatment has emerged as a research focus.

The expression of some miRNAs increases or decreases during the progression of lung cancer, and these miRNAs regulate the expression of coding genes (5). In contrast to other RNAs, miRNAs are endogenous, noncoding, single-stranded small RNAs composed of approximately 20 nucleotides that can regulate the expression of mRNA genes. They can be used to regulate related target genes for tumor therapy because they can always maintain a high degree of conservation (5). Previous research has concentrated on treating lung cancer by regulating a negative regulatory target gene mRNA, a pathway involving miRNA (6); however, some miRNAs can also indirectly positively regulate mRNA by regulating other cytokines (7).

There are thousands of human miRNAs. Previous research on the high expression of miR-21 and low expression of let-7 in lung cancer tissue and cells indicates poor patient survival and promotes tumor progression (8–10). MiR-21 is the most commonly overexpressed miRNA in cancer (11), and it is related to cell proliferation, apoptosis, migration, and invasion (12). It is thought to be a carcinogenic gene because it is involved in tumor promotion. Overexpression of miR-21 removes multiple inhibitors of the RAS/MEK/ERK pathway and promotes tumor progression, whereas knocking down miR-21 inhibits the transformation driven by the RAS gene, thereby inhibiting the development of lung cancer (10, 13). Let-7 is a gene that has been conserved from worms to humans during evolution (14), and it is downregulated in lung cancer (13, 15–17). It is a common RAS family direct negative regulatory factor (13) and can indirectly regulate the target gene K-ras *via* Lin28A/B (18, 19). Let-7 upregulation may slow the growth of lung tumors in mice (20). As a result, in this study, we chose the miR-21 and let-7 genes, which are commonly studied in lung cancer research (13) and predicted that the effect of cooperative regulation of miR-21 and let-7 on lung cancer is more significant than the regulation of a single miRNA alone.

## Materials and methods

### Clinical samples

All lung cancer tissue samples (42 cases) were collected from the Fourth Affiliated Hospital of Harbin Medical University from August 2020 to July 2022 (**Table 1**). The samples were immediately frozen in liquid nitrogen for further analysis. These patients had not received any therapy before sample collection.

**Table 1:** Clinicopathological characteristics.

Characteristics	Data
Total No.	42
Sex (*n* (%))	
Male	21 (50%)
Female	21 (50%)
Age (years (range))	
Mean	60.8 (43–85)
TNM clinical stage (*n* (%))	
I	12 (28.6%)
II	8 (19%)
III	14 (33.4%)
IV	8 (19%)
Smoking status	
Never smoked	8 (19%)
Former smoker	24 (57.1%)
Current smoker	10 (23.9%)

Before collecting clinical samples, all patients provided written informed consent. This study was performed in accordance with the standards established by the Declaration of Helsinki. The study protocol was approved by the Ethics Committee of the Fourth Affiliated Hospital of Harbin Medical University.

### Cell culture and cell transfection

The human lung cancer cell lines A549 and H460 and the human bronchial epithelial cell line HBE were cultured in a 1640 medium containing 10% fetal bovine serum (FBS) at 37°C in a 5% CO_2_ incubator. Using LipofectamineTM 2000 (Lipo2000) as a transfection reagent, a miR-21 inhibitor, miR-21 mimic, let-7 mimic, let-7 inhibitor, NC-inhibitor, and NC-mimic were transfected into A549 and H460 cell lines (**Table 2**). The specific operational procedure of the experiment is based on the Lipofectamine 2000 (Thermo Fisher) (aoheng biotechnology development Co. Ltd. , Harbin City, China) product manual. The sequences of miRNA mimics and miRNA inhibitors are listed in **Table 3**.

**Table 2:** Transient transfection reaction system.

Culture vessel	Amount of inhibitor/mimics	Volume of medium	Volume of reagent
6-Well	2.2 pmol (31 ng)	200 μl	4 ± 2 μl

**Table 3:** Sequences of miRNA mimics and miRNA inhibitors.

Primer	Sequence
MiR-21 mimic	5′-UAGCUUAUCAGACUGAUGUUGA-3′
MiR-21 inhibitor	5′-AUAUCCGCUGAUUCAGCACCAU-3′
Let-7 mimic	5′-AACAGCACAAACUACUACCUCA-3′
Let-7 inhibitor	5′-UGAGGUAGUAGUUUGUGCUGUU-3′

### RNA isolation and qRT-PCR

Total RNA was extracted from lung cancer tissue and cells using the TRIzol reagent. The specific experimental operational procedure of cDNA library construction was carried out according to the product manual of the Transcriptor First-Strand cDNA Synthesis Kit (Roche). All the reaction reagents were melted on ice and mixed lightly. For the SYBR gene detection technology, the specific operation procedure was based on the Roche SYBR-ROX product manual, and the reaction system was prepared according to the operation steps of the kit. The reaction system utilized the 7500 Real-Time PCR instrument (Applied Biosystems), with GAPDH and U6 used as the internal references. The expression level of the qRT-PCR products was calculated by the 2^−ΔΔCt^ method.

RT-PCR primers were designed as follows: K-ras F 5′-TGTGGTAGTTGGAGCTGGTG-3′ and R 5′-TCCAAGAGACAGGTTTCTCCA-3′; pleomorphic adenoma gene 1 (PLAG1) F 5′-ATCACCT CCATACACACGACC-3′ and R 5′-AGCTTGGTATTGTAGTTCTTGCC-3′; β-catenin F 5′-GGAAGGTCTCCTTGGGACTC-3′ and R 5′-ATACCACCCACTTGGCA GAC-3′; miR-21 F 5′-GGGGTAGCTTATCAGACTG-3′ and R 5′-TGGAGTCGGCA ATTGCACTG-3′; let-7 F 5′-TGGAAGACTAGTGATTTTGTTGTT-3′ and R 5′-A TCCAGTGCAGGGTCCGAGG-3′; U6 F 5′-TCCCAGGGCGAGGC TTAT CC ATT-3′ and R 5′-GAACGCAGTCCCCCACTACCACAAA-3′; and GAPDH F 5′-CCCACTCC TCCACCTTTGAC-3′ and R 5′-CATACCAGGAAATGAGCTTGACAA-3′.

### CCK-8 assay

A549 and H460 cells were inoculated into a 96-well plate and incubated at 37°C for 48 h. According to the manufacturer’s instructions, the cell disposal treatment was performed, and the culture medium of each well was changed before adding CCK-8 reagent to each well to reduce the effect of cell metabolites on the assay. Ten microliters of CCK-8 reagent was added to each well to avoid producing air bubbles and affect the assay results. The culture plate was incubated in the incubator for 2 h. The absorbance of each well in the culture plate at 450 nm was determined by a multifunction enzyme labeling instrument, and the data were analyzed by Excel.

### EdU cell proliferation assay

Cells from the logarithmic growth phase were plated in 24-well plates. A total of 100 μl of EdU medium was then added to each well, and the cells were incubated in a 37°C incubator for 2 h. The EdU medium was removed, and the cells were washed with PBS. The cells were fixed with a 4% paraformaldehyde solution at room temperature for 30 min. The formaldehyde fixing solution was removed by pipette, and images were obtained by fluorescence microscopy to analyze the results.

### Western blot

The cell extracts were prepared by lysing cells in RIPA buffer containing a complete protease inhibitor mix (Biyuntian Co. Ltd.). The protein concentration of the cell extracts was measured by a BCA Protein Assay Kit (Biyuntian Co. Ltd.). We separated the molecular weight of the protein samples into different bands by polyacrylamide electrophoresis and then transferred the protein bands on the PAGE gel to the NC membrane. The membranes were treated with anti-PLAG1 antibody (rabbit-derived polyclonal, 1:200, Abcam Corp.) and anti-p-β-catenin antibody (rabbit-derived polyclonal, 1:200, Cell Signaling Technology Corp.) and then incubated with goat anti-mouse secondary antibodies (1:10,000, Alexa Fluor 800). The film was assessed by an infrared fluorescence scanning system. GAPDH (mouse-derived polyclonal, 1:1,000, Santa Cruz Biotechnology) was used as the internal reference. Pictures were taken, and the optical density integral was analyzed by image analysis software Odyssey1.2.

### TUNEL assay

A549 and H460 cells were grown on slides. The TUNEL reaction mixture was prepared according to the instructions from Roche. Fifty microliters of TUNEL reaction mixture was added to each slide, and the slides were placed into a dark wet box and reacted at 37°C for 1 h. The slides were washed twice with PBS for 5 min each time, after which DAPI was added, followed by incubation in an incubator at 37°C for 10 min. The slides were observed and counted under a fluorescence microscope.

### Wound-healing assay

A549 and H460 cells were inoculated into six-well plates and cultured until 100% confluency was achieved. The cells were randomly divided into four groups, which were transfected with miR-21 inhibitor, let-7 mimic, miR-21 inhibitor + let-7 mimic, and control group. Subsequently, a p200 pipette tip was used to create a scratch. A culture medium without FBS was added. The cells were imaged using a microscope at 0 and 48 h after wounding.

### Transwell assay

The A549 and H460 cells were inoculated at the top of the 24-well chamber with a pore diameter of 8 μm that had been precoated with 80 μl Matrigel (BD, China). The lower chamber contained 800 μl of culture medium with 10% fetal bovine serum (FBS). After 24 h of culture, the cells on the upper surface of the chamber were removed with cotton swabs, and the cells invading the lower surface of the chamber were fixed with a 4% paraformaldehyde fixation solution (Beyotime, China). A total of 0.5% crystal violet (Beyotime, China) was then added to stain the cells, which were counted under a microscope (Olympus CKX53, Japan).

### Luciferase assay

The luciferase reporter gene vector containing wild type (WT) and mutant type (MT) was constructed according to the let-7 binding site predicted by starBase v2.0 and TargetScan. The control mimic and let-7 mimic were synthesized and cotransfected with the luciferase reporter gene and gene fragment into H460 cells by the transfection reagent. The luciferase activities were measured with the luciferase reporter assay system.

### Statistical analysis

Data analysis was performed with SAS 9.4 software and GraphPad Prism software (GraphPad Software, La Jolla, CA, USA). Values are presented as the mean ± standard deviation (SD). The Student’s *t*-test was used for statistical analysis. A paired *t*-test was used to assess the expression of miRNAs in lung cancer and paracancerous tissues. The three groups of cell lines were first analyzed by one-way ANOVA, and the difference was statistically significant. Dunnett’s *t*-test was then performed. Factorial design ANOVA and individual effect comparison were used to analyze the differences between regulatory groups. A one-sample *t*-test was used to analyze the differences between the NC group and the regulatory group. A *p* < 0.05 (two-tailed) was considered a significant difference. All experiments were repeated in triplicate at a minimum.

## Results

### Expression of miR-21 and let-7 in lung cancer tissues and cells

In this study, 42 patients with lung cancer were analyzed, with an average age of 60.8 years (43-85). The male-to-female ratio was 1:1, with TNM stages of I–IV (**Table 1**).

**Figure 1:**
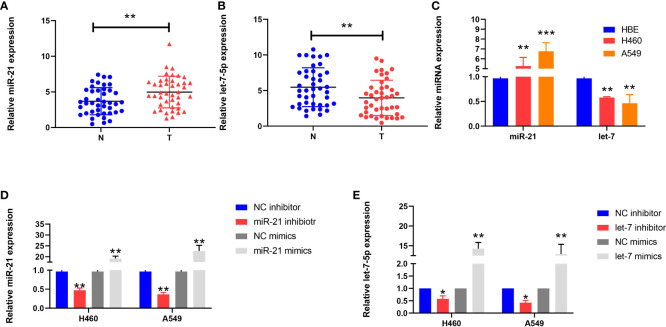
Expression of miR-21 and let-7 in lung cancer cells. **(A, B)** Relative expression of miR-21 and let-7 in lung cancer and paracancerous tissues. **(C)** Relative expression of miR-21 and let-7 in lung cancer cell lines (H460 and A549) and the normal cell line HBE. **(D, E)** Relative expression of miR-21 and let-7 after transfection of miR-21/let-7 inhibitors or mimics (all qRT-PCR data are represented as the means ± SDs of three independent experiments; ^*^ *p* < 0.05; ^**^ *p* < 0.01; ^***^ *p* < 0.001).

We used qRT-PCR to analyze the expression of miR-21 and let-7 in 42 cases of lung cancer. The results showed that the expression of miR-21 was significantly higher than that in paracancerous tissues (4.985 ± 2.226 vs. 3.695 ± 1.897; *p* = 0.0015; **Figure 1A**); in contrast, let-7 showed a low expression (3.969 ± 2.471 vs. 5.468 ± 2.720; *p* = 0.001; **Figure 1B**). Similar results were obtained in cell lines. Compared with human normal bronchial epithelial cells (HBEs), the H460 and A549 cell lines showed a high expression of miR-21 (*p* = 0.001 and *p* <0.001, respectively) and a low expression of let-7 (*p* = 0.004 and *p* = 0.001, respectively) (**Figure 1C**). This finding is consistent with previous studies on the high expression of miR-21 and the low expression of let-7 in lung cancer (11, 13, 15, 16).

### Expression of miR-21 and let-7 in lung cancer cells after regulation

After transfection of the miR-21 inhibitor into H460 and A549 cells, the transfection efficiency was measured by qRT-PCR assay. The expression level of miR-21 was lower than that of the control group (*p* = 0.0038 and *p* = 0.0023, respectively), but the expression of miR-21 increased after transfection of the miR-21 mimic (*p* = 0.0075 and *p* = 0.0098, respectively) (**Figure 1D**). The same results were obtained after transfection with a let-7 inhibitor and mimic (**Figure 1E**). This demonstrates that the two miRNAs can be stably expressed in cells.

### Proliferation of lung cancer cells after miRNA regulation

**Figure 2:**
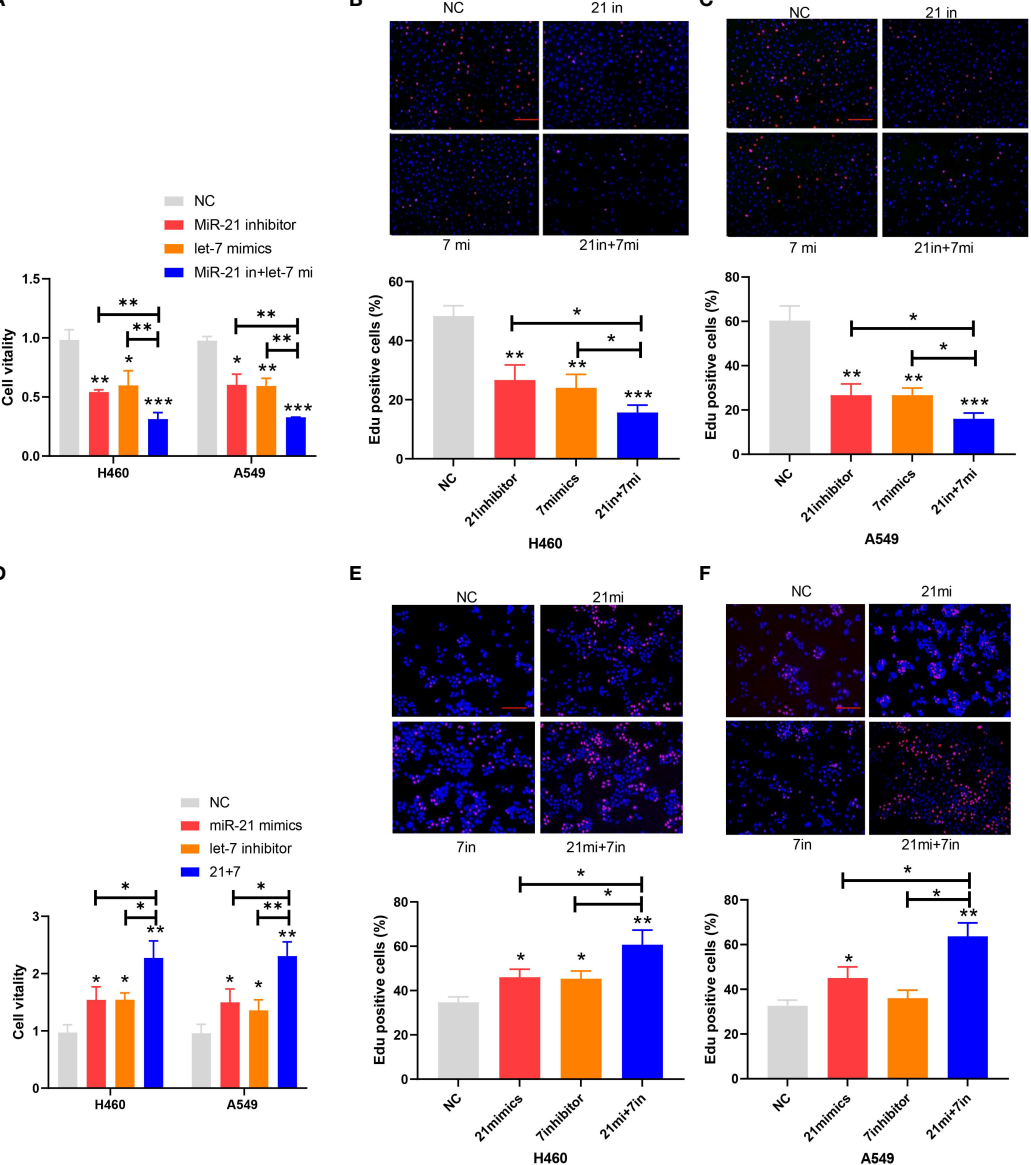
Proliferation of lung cancer cell lines. **(A–C)** Cell viability after transfection of the miR-21 inhibitor and let-7 mimic in lung cancer cell lines. **(D–F)** EdU cell proliferation after transfection of the miR-21/let-7 inhibitors or mimics in lung cancer cell lines (scale bar: 100 μm; *p < 0.05; **p < 0.01; ***p < 0.001).

The proliferation of cancer cells in every group of cell lines was analyzed by the CCK-8 and EdU cell proliferation assays. The inhibitory rate of miR-21 on tumor growth in the let-7 mimic group compared to the NC group was 43.65% and 41.27%, respectively, and that of the 21 in+7 mimic group showed an average value of 69.32% for the two groups of cell lines (*n* = 3, *p* < 0.001) (**Figures 2A–C**). Upregulation of miR-21 and downregulation of let-7 promoted the proliferation of lung cancer cells, especially in the 21 mimic group, which was 1.54 times that of the NC group. The effect of cooperative regulation of the two kinds of miRNA was 2.37 times that of the NC group in promoting the proliferation of lung cancer cells (**Figures 2D–F**).

### Apoptosis of lung cancer cells after miRNA regulation

**Figure 3:**
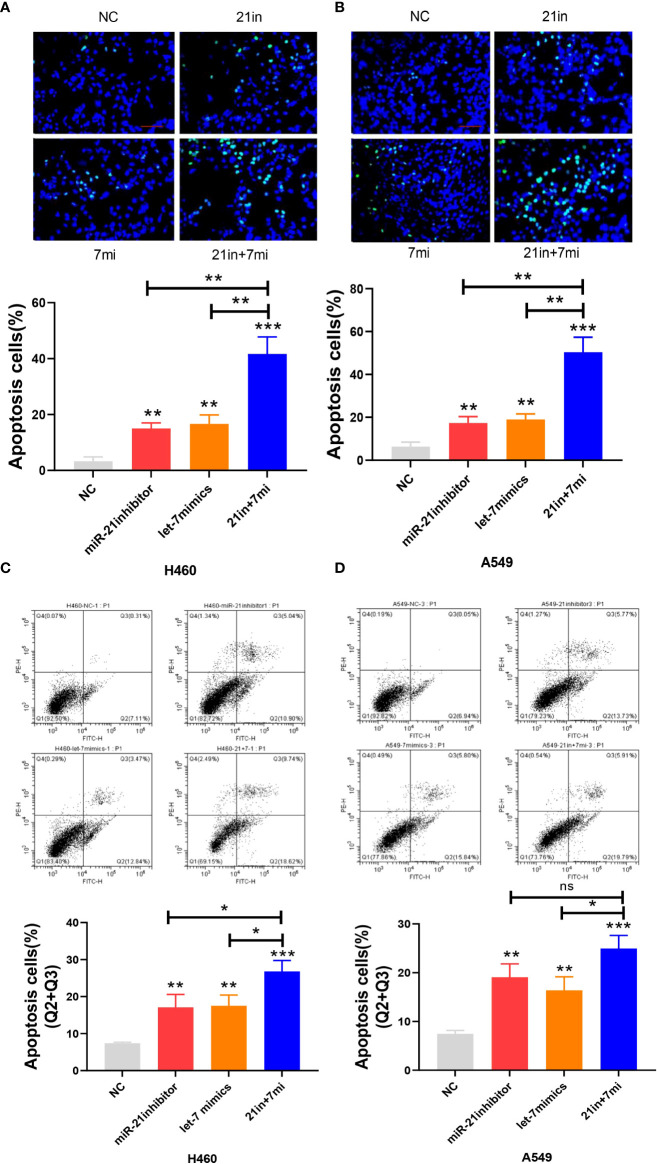
Apoptosis assay of lung cancer cell lines. **(A, B)** The apoptosis of cells as assessed by the TUNEL assay after transfection of miR-21/let-7 inhibitors or mimics in lung cancer cell lines. **(C, D)** Results of the flow cytometry assay after transfection with miR-21/let-7 inhibitors or mimics in lung cancer cell lines (scale bar: 100 μm; ns, no significance; *p < 0.05; **p < 0.01; ***p < 0.001).

**Figure 4:**
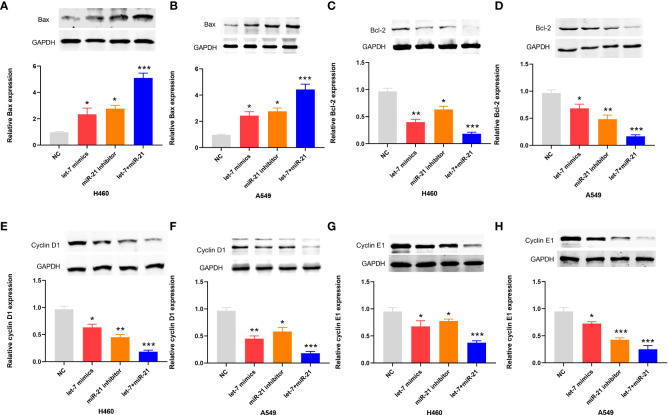
Western blot results of apoptotic proteins in lung cancer cell lines. **(A–H)** The relative levels of Bax, Bcl-2, cyclin D1, and cyclin E1 expression after transfection of miR-21/let-7 inhibitors or mimics in lung cancer cell lines (*p < 0.05; **p < 0.01; ***p < 0.001)..

In the apoptosis experiment, the TUNEL incorporation assay showed that the proportion of apoptotic cells in the miR-21 inhibitor group and let-7 mimic group was three to five times higher than that in the control group, indicating that miR-21 inhibited tumor cell apoptosis and let-7 promoted tumor cell apoptosis, while the effect of cooperative regulation of the two kinds of miRNAs was greater (**Figures 3A, B**). The flow cytometry results in the Q2 and Q3 quadrants also showed similar results (**Figures 3C, D**). Furthermore, we demonstrated changes in the expression of Bax, Bcl-2, cyclin D1, and cyclin E1 by Western blotting (21–23), which supported the above results. The Bax protein expression in the miR-21 inhibitor group and let-7 mimic group was higher than that in the control group, especially in the miR-21 inhibitor+let-7 mimic group (*n* = 3, *p* < 0.001, **Figures 4A, B**). The expression of Bcl-2, cyclin D1, and cyclin E1 was lower than that in the control group, and the expression in the miR-21 inhibitor+let-7 mimic group was more obvious (*n* = 3, *p* < 0.001, **Figures 4C–H**).

### Migration and invasion of lung cancer cells after miRNA regulation

**Figure 5:**
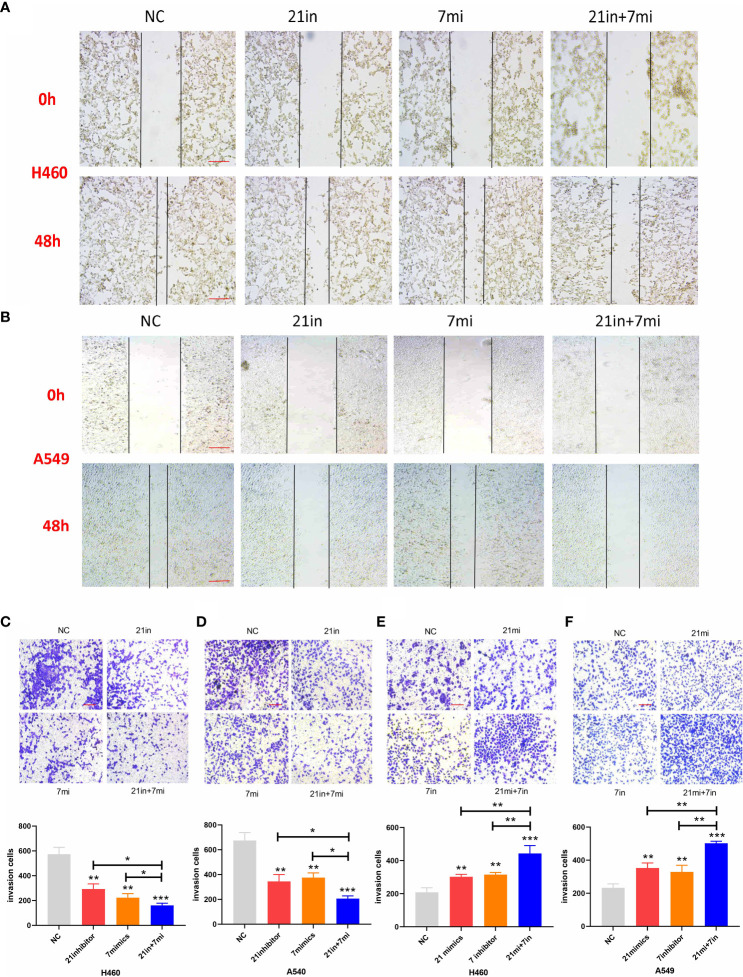
Wound-healing and Transwell assays of lung cancer cell lines. **(A, B)** Relative migrating areas after transfection of miR-21/let-7 inhibitors or mimics at 0 and 48 h. **(C–F)** Relative invasion areas after transfection of miR-21/let-7 inhibitors or mimics in lung cancer cell lines (scale bar: 100 μm; *p < 0.05; **p < 0.01; ***p < 0.001).

Downregulation of miR-21 and upregulation of let-7 inhibited the migration and invasion of lung cancer cells in wound-healing and Transwell assays, and the effect of combined regulation was better than that of single regulation (**Figures 5A–D**). Upregulation of miR-21 and downregulation of let-7 promoted the invasion of lung cancer cells in the Transwell assay (*p* < 0.01), and the effect of regulating both miRNAs was more obvious (*p* < 0.001, **Figures 5E, F**).

### Expression of β-catenin after regulating miR-21 and the correlation between miR-21 and β-catenin

**Figure 6:**
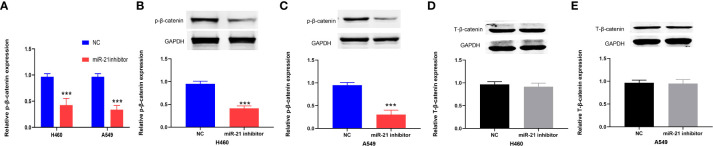
miR-21 is positively correlated with p-β-catenin but not T-β-catenin in lung cancer cells. **(A–C)** Western blot analysis of the expression of p-β-catenin in H460 and A549 cells treated with miR-21 inhibitor. **(D, E)** The expression level of T-β-catenin in H460 and A549 cells transfected with miR-21 inhibitor (***p < 0.001).

The Wnt/β-catenin pathway plays an important role in the progression of cancers, including lung cancer (24). Previous research has shown that the Wnt/β-catenin signaling pathway is positively correlated with miR-21 in lung cancer (25). Therefore, we divided the cells in this experiment into a miR-21 inhibitor group and a control group. qRT-PCR was used to determine the expression of β-catenin in lung cancer cells (H460 and A549) in the miR-21 inhibitor group. Western blot experiments were carried out with GAPDH as an internal reference to observe the protein expression of β-catenin. The results showed that the expression of phosphorylated β-catenin in the two kinds of lung cancer cells in the miR-21 inhibitor group was significantly lower than that in the control group. (*p* < 0.001, **Figures 6A–C**), but the expression of total β-catenin (T-β-catenin) was not inhibited (*p* = 0.7953, **Figures 6D, E**). It has been proven that p-β-catenin is a key molecule for miR-21. In previous studies, the expression of T-β-catenin was not significantly changed after the downregulation of miR-21, but the relationship between p-β-catenin and miR-21 was not analyzed (25).

### Expression of PLAG1 after regulating let-7 and the correlation between let-7 and PLAG1

**Figure 7:**
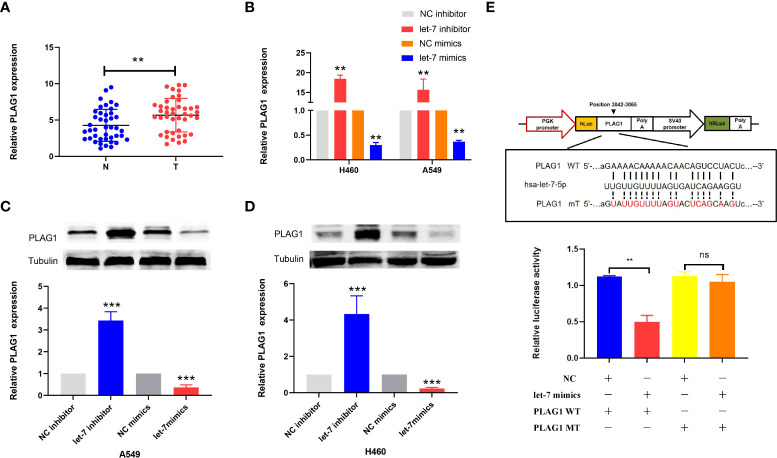
The expression of PLAG1 in lung cancer tissue and the relationship between let-7 and PLAG1 as demonstrated by Western blotting and luciferase assays in cells. **(A)** The expression of PLAG1 in lung cancer tissue was higher. **(B–D)** The relative PLAG1 expression after transfection of let-7 inhibitor or mimics in lung cancer cell lines. **(E)** The binding sites of let-7 and PLAG1 and the relative luciferase activity of the let-7 mimic cotransfected with WT/MT were evaluated (ns, no significance; **p < 0.01; ***p < 0.001)..

By using miRBase and TargetScan, it was predicted that the target of let-7 may be PLAG1. We used qRT-PCR to determine the expression of PLAG1 in 42 cases of lung cancer. The results showed that the expression of PLAG1 in lung cancer tissues was significantly higher than that in adjacent tissues (the difference between the means was 1.408 ± 0.4914, *p* = 0.0053, **Figure 7A**). Similar results were obtained in cell lines (*p* < 0.01, **Figure 7B**). The cells were divided into the let-7 inhibitor group, let-7 mimic group, and control group. Western blotting was then carried out using tubulin as an internal reference to observe the protein expression of PLAG1 in H460 and A549 cells. The results of the Western blot assay showed that in the two kinds of lung cancer cells, the expression of PLAG1 in the let-7 inhibitor group was higher than that in the control group, while the expression of PLAG1 in the let-7 mimic group was decreased (*p* < 0.001, **Figures 7C, D**), which proved that PLAG1 was regulated by let-7. To further verify that PLAG1 was the direct target of let-7 and to demonstrate the binding site between let-7 and PLAG1, we carried out a luciferase experiment. The results showed that let-7 inhibited the expression of the luciferase reporter gene when transfected with PLAG1 MT in HEK293T cells but had no inhibitory effect when transfected with PLAG1 MT and NC (**Figure 7E**).

### Expression of K-ras and the correlation of K-ras to miR-21 and let-7 after regulating K-ras

**Figure 8:**
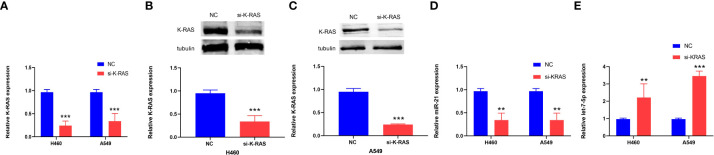
The expression of K-ras in cells and the relationship to miR-21 and let-7. **(A–C)** qRT-PCR and Western blot analyses showing downregulation of K-ras following transfection with K-ras siRNA into H460 and A549 cells. **(D, E)** qRT-PCR results showing that downregulation of K-ras in H460 and A549 cells resulted in a decrease in the expression of miR-21 and an increase in let-7 compared with the corresponding normal control group (**p < 0.01; ***p < 0.001).

Si-K-ras were introduced into lung cancer cells (H460 and A549) to knock down K-ras, resulting in a significant decrease in gene expression by qRT-PCR (*p* < 0.001, **Figure 8A**). Further research showed that when tubulin was used as a reference for Western blot experiments, the protein expression of K-ras was decreased in the two kinds of lung cancer cells (*p* < 0.001, **Figures 8B, C**). After the downregulation of K-ras, the expression of miR-21 in lung cancer cells decreased, and the expression of let-7 increased (*p* < 0.01 and *p* < 0.001, **Figures 8D, E**). It is suggested that K-ras positively regulates miR-21, while K-ras negatively regulates let-7.

## Discussion

At present, the high incidence and low survival rate of lung cancer is a common problem for humans (26). There are many endogenous and exogenous reasons, and the most fundamental reason is tumor heterogeneity (27), which makes it difficult to treat. MiR-21 can promote the growth and migration of lung cancer cells as an oncogene (28). It has been proven that miR-21 inhibitors can suppress the development of lung cancer (29). In contrast, Let-7 is an important tumor suppressor gene discovered in recent years. Let-7 mimics can induce apoptosis of lung cancer cells and reduce tumor cell invasiveness (30). According to previous research results, we found that the expression of miR-21 in lung cancer tissues and cells was upregulated, while the expression of let-7 was downregulated. This shows that miR-21 and let-7 are involved in the occurrence and development of lung cancer, which is consistent with the report of Choudhury et al. (13).

The activation of β-catenin promotes the proliferation and survival of lung cancer cells, induces angiogenic factors that promote tumor angiogenesis, and maintains cell integrity (31). Downregulation of miR-21 leads to the downregulation of p-β-catenin, which plays an inhibitory role in lung cancer. We found that upregulation of let-7 suppressed the expression of PLAG1, while PLAG1 could promote apoptosis resistance and metastasis of lung cancer by regulating glutamate dehydrogenase 1 (GDH1) (32). Studies have shown that GDH1 promotes tumor growth by activating the reactive oxygen species (ROS)-scavenging enzyme glutathione peroxidase 1 and regulating redox homeostasis through its product a-ketoglutarate (a-KG) and the subsequent metabolite fumarate (32). The binding site of let-7 and PLAG1 was verified by luciferase experiments in our experiment. Therefore, upregulation of let-7 suppressed PLAG1, which targets GDH1 to inhibit lung cancer cells.

Downregulation of miR-21 and upregulation of let-7 at the same time showed a more obvious antitumor effect, indicating that there is a feedback regulation loop between miR-21 and let-7 to coordinate the regulation of lung cancer. We found that knocking down K-ras can inhibit the expression of miR-21 and promote the expression of let-7, and therefore, K-ras may be a common target gene involved in the synergistic regulation of miR-21 and let-7 in lung cancer. Previous studies have shown that miR-21 indirectly positively regulates RAS genes through multiple negative regulators of the RAS pathway, of which Spry1, Spry2, Btg2, and Pdcd410 have been confirmed, and miR-21 also positively regulates the downstream gene AP1 of K-ras by inhibiting Spry1/2 or Pdcd4 (13, 33, 34). In contrast, it has been proven that let-7 can directly inhibit K-ras expression within the binding site of the K-ras 3′UTR (13, 35, 36) or indirectly by inhibiting LIN28A/B (13, 17). These reports support our research that miR-21 and let-7 participate in the regulation of lung cancer through K-ras.

**Figure 9:**
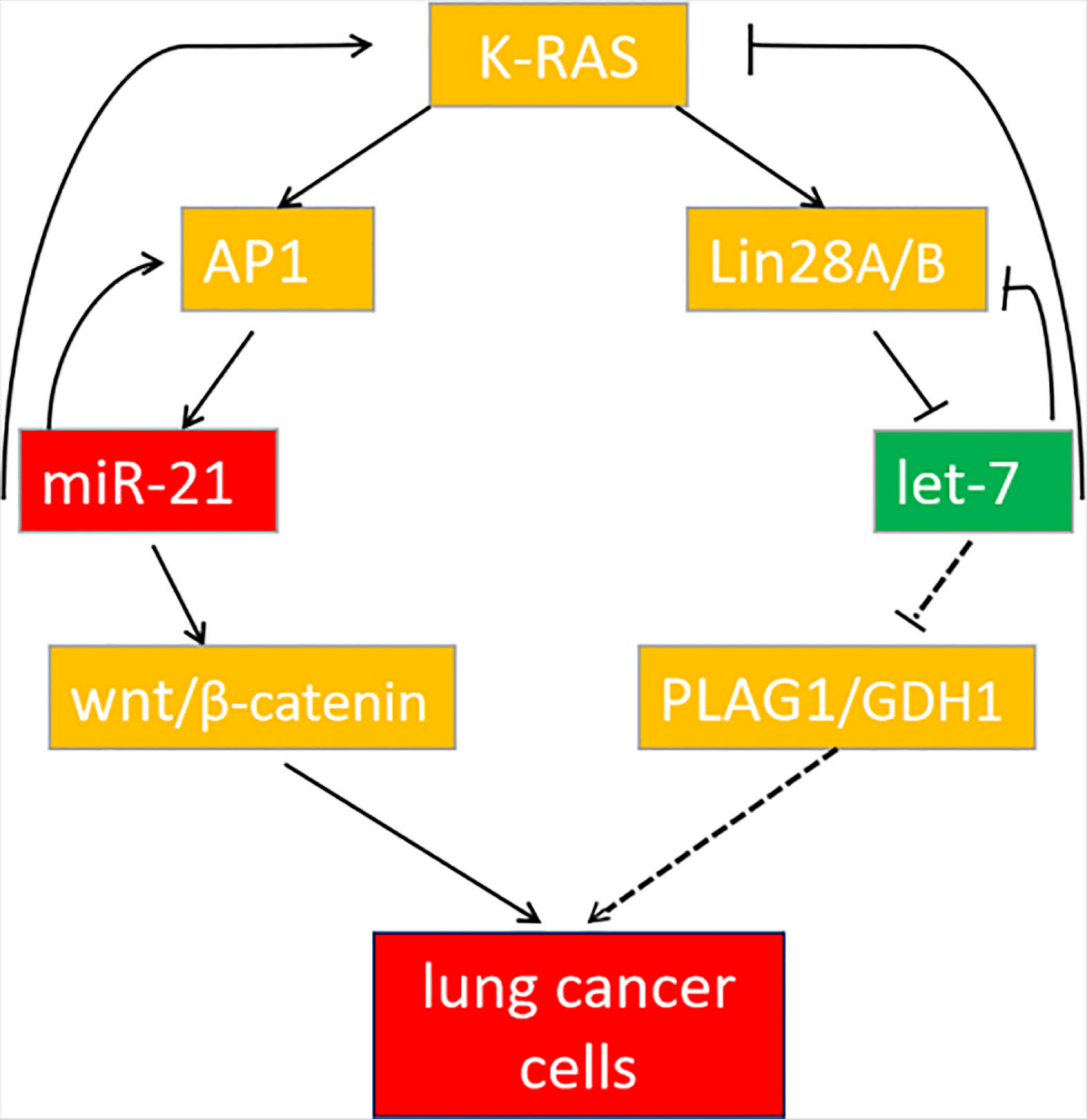
The feedback pathway between miR-21 and let-7. The arrow indicates positive regulation, and the horizontal line indicates negative regulation.

In summary, miR-21 and let-7 are important differentially expressed genes in lung cancer tissues and cells. Downregulation of miR-21 or upregulation of let-7 can both inhibit the development of lung cancer, but the cooperative regulation of miR-21 and let-7 exerts a more significant effect. These genes participate in the regulation of lung cancer through the K-ras gene to form a feedback pathway (**Figure 9**). For multigene-involved lung cancer, cooperative regulation of the two miRNAs will provide new targets and directions for lung cancer treatment in the future.

## Data availability statement

The datasets presented in this study can be found in online repositories. The names of the repository/repositories and accession number(s) can be found in the article/supplementary material.

## Ethics statement

The studies involving human participants were reviewed and approved by the Ethics Committee of the Fourth Affiliated Hospital of Harbin Medical University. The patients/participants provided their written informed consent to participate in this study. Written informed consent was obtained from the individual(s) for the publication of any potentially identifiable images or data included in this article.

## Author contributions

TZ designed the study. JB, ZS, SW, and HP performed the experiments and analyzed the data. JB contributed to drafting the manuscript. All authors read and approved the final manuscript.

## Funding

This work was supported by the Beijing Cihua Medical Development Foundation Project (Research on CT-assisted diagnosis of coronary heart disease based on artificial intelligence).

## Conflict of interest

The authors declare that the research was conducted in the absence of any commercial or financial relationships that could be construed as a potential conflict of interest.

## Publisher’s note

All claims expressed in this article are solely those of the authors and do not necessarily represent those of their affiliated organizations, or those of the publisher, the editors and the reviewers. Any product that may be evaluated in this article, or claim that may be made by its manufacturer, is not guaranteed or endorsed by the publisher.
