# Corrigendum: MiR-21 and let-7 cooperation in the regulation of lung cancer

**DOI:** 10.3389/fonc.2025.1602462

**Published:** 2025-05-29

**Authors:** Jinquan Bai, Zhenzhou Shi, Shuting Wang, Hong Pan, Tong Zhang

**Affiliations:** Department of Radiology, The Fourth Affiliated Hospital of Harbin Medical University, Harbin, China

**Keywords:** lung cancer, miR-21, let-7, K-ras, cooperative regulation

In the published article, there was an error in **Figure 2** as published. Recently, when sorting out the previously published articles, we found that the E, F pictures in **Figure 2** of this paper are obviously different from the B, C pictures. Our investigation found that the reason was the wrong storage path of E, F pictures, which led to this kind of low-level error. The corrected **Figure 2** and its caption appear below.

**Figure 2:**
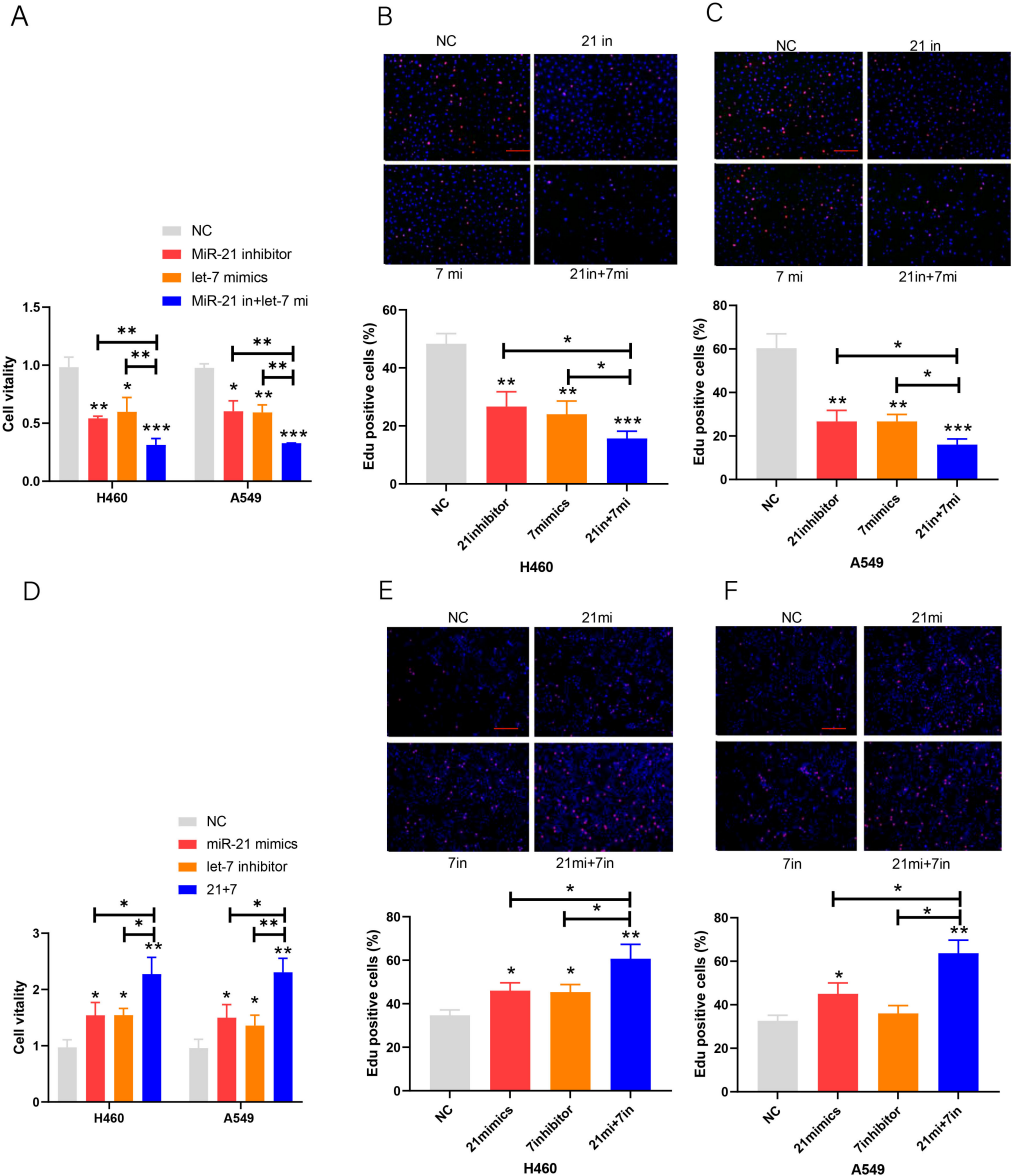
Proliferation of lung cancer cell lines. **(A–C)** Cell viability after transfection of the miR-21 inhibitor and let-7 mimic in lung cancer cell lines. **(D–F)** EdU cell proliferation after transfection of the miR-21/let-7 inhibitors or mimics in lung cancer cell lines (scale bar: 100 μm; *p < 0.05; **p < 0.01; ***p < 0.001).

The authors apologize for this error and state that this does not change the scientific conclusions of the article in any way. The original article has been updated.

